# Decreased Brain pH Correlated With Progression of Alzheimer Disease Neuropathology: A Systematic Review and Meta-Analyses of Postmortem Studies

**DOI:** 10.1093/ijnp/pyae047

**Published:** 2024-10-18

**Authors:** Hideo Hagihara, Tsuyoshi Miyakawa

**Affiliations:** Division of Systems Medical Science, Center for Medical Science, Fujita Health University, Toyoake, Japan; Division of Systems Medical Science, Center for Medical Science, Fujita Health University, Toyoake, Japan

**Keywords:** Alzheimer disease, brain pH, Braak stage, systematic review, meta-analysis

## Abstract

**Background:**

Altered brain energy metabolism is implicated in Alzheimer disease (AD). Limited and conflicting studies on brain pH changes, indicative of metabolic alterations associated with neural activity, warrant a comprehensive investigation into their relevance in this neurodegenerative condition. Furthermore, the relationship between these pH changes and established AD neuropathological evaluations, such as Braak staging, remains unexplored.

**Methods:**

We conducted quantitative meta-analyses on postmortem brain and cerebrospinal fluid pH in patients with AD and non-AD controls using publicly available demographic data. We collected raw pH data from studies in the NCBI GEO, PubMed, and Google Scholar databases.

**Results:**

Our analysis of 20 datasets (723 patient samples and 524 control samples) using a random-effects model showed a significant decrease in brain and cerebrospinal fluid pH in patients compared with controls (Hedges’ *g *= −0.57, *P* < .0001). This decrease remained significant after considering postmortem interval, age at death, and sex. Notably, pH levels were negatively correlated with Braak stage, indicated by the random-effects model of correlation coefficients from 15 datasets (292 patient samples and 159 control samples) (adjusted *r *= −0.26, *P *< .0001). Furthermore, brain pH enhanced the discriminative power of the *APOE*ε4 allele, the most prevalent risk gene for AD, in distinguishing patients from controls in a meta-analysis of 4 combined datasets (95 patient samples and 87 control samples).

**Conclusions:**

The significant decrease in brain pH in AD underlines its potential role in disease progression and diagnosis. This decrease, potentially reflecting neural hyperexcitation, could enhance our understanding of neurodegenerative pathology and aid in developing diagnostic strategies.

**Significance Statement**

The observed decrease in brain and cerebrospinal fluid pH levels in Alzheimer disease (AD), as revealed by our comprehensive meta-analysis, provides crucial insights into the neurodegenerative processes underlying this condition. Our findings, based on extensive data analysis of 723 AD patient samples and 524 control samples, demonstrate a significant association between altered pH and AD pathology. Notably, the negative correlation between pH levels and Braak stage further underscores the relevance of pH changes in disease progression. Importantly, our study establishes the potential utility of brain pH as an adjunctive diagnostic marker, enhancing the discriminatory power of the *APOE*ε4 allele, a prominent risk gene for AD. This perspective on metabolic alterations and decreased brain pH suggests a shared endophenotype across AD and other neuropsychiatric disorders, offering new avenues for understanding and targeting common mechanisms in these conditions.

## INTRODUCTION

Alzheimer disease (AD) is a prevalent neurodegenerative disease worldwide, posing one of the most significant health burdens of the 21st century (Prince et al., 2015; Scheltens et al., 2016). Genetic studies have demonstrated that AD has a high heritability, with multiple genetic factors contributing to the disease. Over 50 risk loci have been associated with AD at a genome-wide significance level (Sims et al., 2020). Among them, point mutations leading to the ε4 allele of apolipoprotein E (*APOE*) are known to be the most potent genetic risk factor for the common sporadic and late-onset forms of AD, as well as influencing rarer familial and early-onset forms of the disease (Saunders et al., 1993; Belloy et al., 2019; Sims et al., 2020; Serrano-Pozo et al., 2021). While the *APOE* gene is suggested to have no association with disease progression (Wilkosz et al., 2010), many other factors can influence the development and progression of pathophysiological changes in the brains of individuals with AD. In particular, pH in the brain could be significant in the pathophysiology of AD, as low pH has been reported to exacerbate the aggregation of the amyloid-β peptide and hyperphosphorylation of Tau protein, key pathological hallmarks of the disease (Atwood et al., 1998; Basurto-Islas et al., 2013; Decker et al., 2021). To date, studies of pH changes in the brain of AD have been limited and results are inconsistent. One postmortem study involving 4 available datasets has reported a decrease in brain and cerebrospinal fluid (CSF) pH in patients with AD compared with control subjects in each dataset (Decker et al., 2021). The results of the magnetic resonance spectroscopy (MRS) studies were inconsistent: a decrease (Lyros et al., 2020), no significant change (Mandal et al., 2012), and an increase (Mecheri et al., 1997; Rijpma et al., 2018) in pH have been observed in the hippocampus and other regions of the brain of patients with AD. Furthermore, no study has yet elucidated the relationship between brain pH and the progression of AD, particularly in the context of Braak staging—a well-established neuropathological method for AD diagnosis based on neurofibrillary tangles (Braak and Braak, 1991).

Decreased brain pH has been consistently suggested in schizophrenia and bipolar disorder by meta-analysis of MRS and postmortem studies (Dogan et al., 2018; Hagihara et al., 2018; Pruett and Meador-Woodruff, 2020). We recently found a similar phenomenon in major depressive disorder in a systematic review and meta-analysis of postmortem studies (Hagihara and Miyakawa, unpublished data). A decrease in brain pH is suggested to be associated with an increase in lactate level under these psychiatric disorders (Prabakaran et al., 2004; Stork and Renshaw, 2005; Halim et al., 2008), which could be due to metabolic changes resulting from mitochondrial dysfunction and/or increased glycolysis due to neuronal hyperexcitation. An increase in brain (Mullins et al., 2018; Hirata et al., 2024) and CSF (Liguori et al., 2015, 2016) lactate levels has been observed in patients with AD compared with control subjects, suggesting common metabolic alterations and neuronal hyperexcitability across such psychiatric and neurodegenerative disorders.

In view of the limited studies and contradictory results regarding pH changes in AD, in this study we conducted a systematic review and quantitative meta-analyses of pH in the postmortem brain and CSF in patients with AD compared with non-AD control subjects, utilizing publicly available demographic data. Furthermore, we conducted meta-analyses to investigate the association between pH and disease progression assessed by the Braak staging and the discriminative ability of brain pH in distinguishing patients with AD from control subjects.

## METHODS

### Ethics Approval and Consent to Participate

This study was performed based on publicly available data, and no separate ethical approval was required.

### Identification and Selection of Eligible Datasets of Postmortem Brain and CSF pH

**Figure 1.**
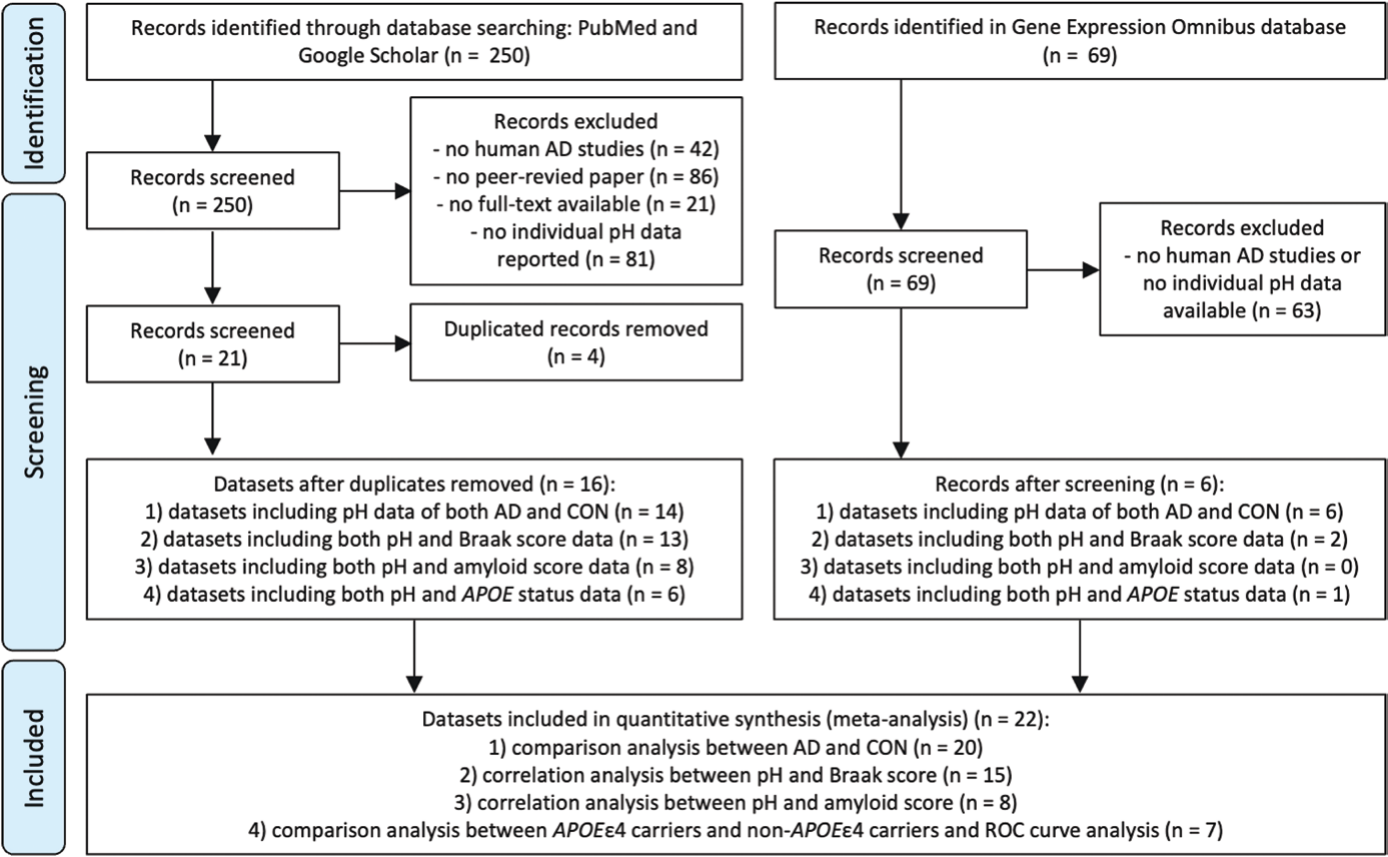
Workflow of the selection of postmortem brain and CSF pH datasets.

Datasets were selected based on the availability of raw pH data of individual subjects. We searched the National Center for Biotechnology Information (NCBI), Gene Expression Omnibus database (GEO), PubMed, and Google Scholar for studies reporting individual pH data with key words “Alzheimer’s disease,” “brain pH,” “postmortem,” and “Braak.” The dataset searches followed the Preferred Reporting Items for Systematic Reviews and Meta-Analyses guidelines (Page et al., 2021) and were conducted on October 2023. The workflow of the selection of pH datasets is shown in Figure 1. Articles were screened first based on their titles, followed by a subsequent evaluation of their abstracts to determine eligibility for full-text review. For records identified in PubMed and Google Scholar, inclusion criteria were of studies involving human AD vs non-AD control comparisons (CON) using postmortem brain or CSF samples and the availability of full text and individual raw data. To minimize the inclusion of overlapping samples across different studies, we attempted to identify and remove duplicated samples from studies that used partially overlapping samples from the same source and then combined the remaining data into a single dataset. If conditions other than CON or AD were present within the study, they were excluded from the current analysis. We also obtained the accompanying demographic information from the identified datasets (i.e., postmortem interval [PMI], age at death, sex, Braak stage, and *APOE* genotype). In the dataset of Ueberham 2006, Braak stage labeled as V−VI was treated as 5.5. In the datasets that provided ABC score, scores 1, 2, and 3 in category “B” (representing Braak stage) were treated as stages 1.5, 3.5, and 5.5, respectively, as score 1 encompasses Braak stage I or II, score 2 corresponds to stage III or IV, and score 3 to stages V or VI (Montine et al., 2012). A score of 0 indicates the absence of neurofibrillary pathology. In the dataset of Gabitto 2023, age labeled as 90+ years was considered as 90.

### Data Analysis

Effect size, measured by Hedges’ *g*, and standard error were calculated within each dataset, and the random-effects model was applied using the function metagen in the R package meta (version 6.5-0). ANCOVA were performed using the GLM procedure in SAS (version 3.81; SAS Institute Inc., Cary, NC, USA). Since the datasets GSE44771, GSE44770, and GSE44768 were derived from the same subjects, GSE44771 was included as a representative in a 2-way ANCOVA to avoid duplication of accompanying demographic information (i.e., age, sex, and PMI). For a correlation analysis, we calculated the Pearson correlation coefficient within each dataset. To estimate the standard error of the correlation coefficient, we used the formula: SE_*r*_ = (1−*r*^2^)/(*n*−2)^1/2^, where *r* is the Pearson correlation coefficient, and *n* is the number of samples in the correlation. We then applied the random-effects model to the correlation coefficient (*r*) and its standard error (SE_*r*_), as described above. Z-score transformation, a method of data normalization for direct comparison between different conditions, was applied to each pH value using individual subject data within each dataset according to the following formula: Z-score = (value_P_—mean value_P1 . . . Pn_)/SD_P1 . . . Pn_, where P is any pH and P1 . . . Pn represent the aggregate measure of all pH values. We conducted the receiver operating characteristics (ROC) curve analysis to evaluate the discriminative ability of the *APOE*ε4 status, pH level, and their combination in identifying patients with AD from control subjects, as assessed by the numerical gains in the area under the curve (AUC). The ROC analyses were performed using GraphPad Prism 8 (version 8.4.2; GraphPad Software, San Diego, CA, USA). For ROC analyses, z-score transformation, a conventional method for data normalization to enable direct comparison between different conditions, was applied to pH values within each dataset, given their heterogeneity across datasets. Multiple logistic regression analyses were applied to the z-score of pH level, *APOE*ε4 status (yes = 1, i.e., ε3/ε4 and ε4/ε4; no = 0), and their combination, respectively. Subgroup analyses of pH levels based on sample types and brain regions were conducted using z-score–transformed values from the respective datasets that reported this information.

## RESULTS

### Search Results

**Figure 2.**
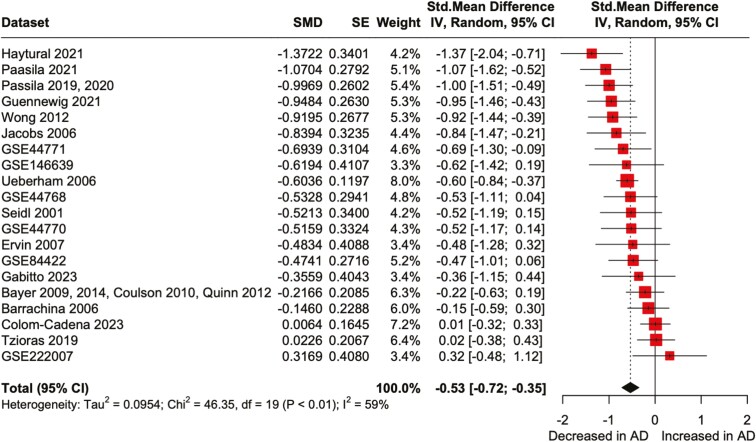
Decreased pH in the postmortem brain and CSF of patients with AD. Forest plot of meta-analysis comparing postmortem brain and CSF pH between patients with AD and non-AD control subjects. Abbreviations: 95% CI, 95% confidence interval; SMD, standardized mean difference.

**Figure 3.**
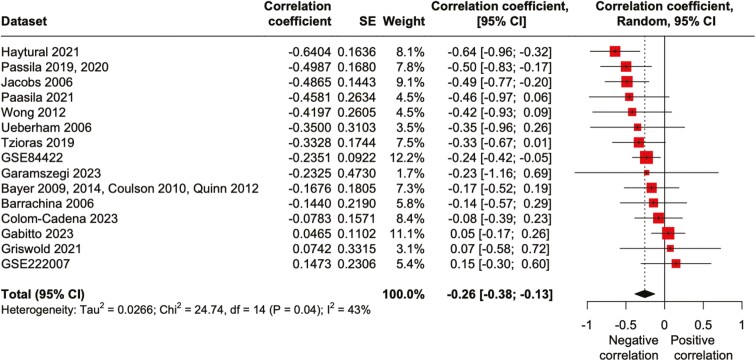
Negative correlation between brain pH and Braak stages. Forest plot of meta-analysis of correlation between brain pH and Braak stages. Abbreviations: 95% CI, 95% confidence interval; CC, correlation coefficient.

**Figure 4.**
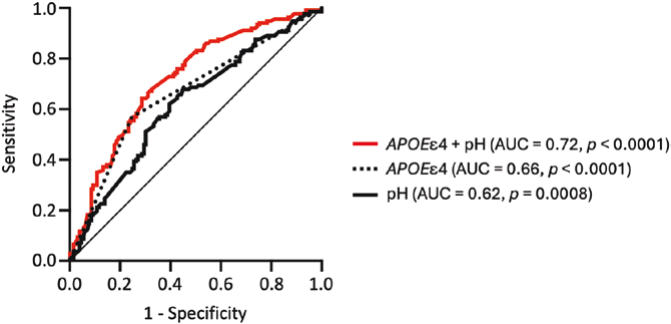
Brain pH level increases discriminative accuracy of *APOE*ε4 status for AD. ROC curves for brain pH level, *APOE*ε4 status, and their combination to assess the accuracy of prediction of AD against controls. Abbreviation: AUC, area under the curve.

Following screening the 250 and 67 records retrieved from the searches in PubMed and Google Scholar, and the NCBI GEO database, 20 and 4 studies were identified to report raw pH data, respectively (Figure 1). After removing duplicates, a total of 22 datasets were processed for the meta-analysis: (1) 20 datasets for comparison analysis of pH between patients with AD and control subjects (Figure 2); (2) 15 for correlation analysis between pH and Braak stage (Figure 3); (3) 8 for correlation analysis between pH and score for amyloid pathology (supplementary Figure 5); and (4) 7 for comparison analysis of pH between *APOE*ε4 carriers and non-*APOE*ε4 carriers (supplementary Figure 7) and ROC curve analysis (Figure 4). A summary of the datasets and more detailed information on pH, PMI, age at death, and sex are presented in Table 1 and supplementary Table 1. The source and references of the datasets used are also included in supplementary Table 1. The raw data analyzed in this study are provided in supplementary Table 2.

**Table 1.** Overall Characteristics of the Datasets Used in This Study

Group	No. of subjects (female/male)	No. of samples	Age (y)^*a*^	Postmortem interval (h)^*a*^	pH^*b*^
CON	360^*c*^ (153/200)	525	79.86 ± 0.71	22.91 ± 1.11	6.54 ± 0.016
AD	516^*d*^ (299/207)^*e*^	739	82.58 ± 0.50^*f*^	17.87 ± 1.01^*f*^	6.37 ± 0.012^*f*^

^*a*^Mean ± SEM based on the number of subjects.

^*b*^Mean ± SEM based on the number of samples.

^*c*^Gender information was unavailable for 7 subjects.

^*d*^Gender information was unavailable for 10 subjects.

^*e*^ *P* < .01, chi-square test.

^f^ *P < *.01, unpaired *t* test.

### Decreased Brain and CSF pH in AD

In the 20 datasets obtained (supplementary Table 1), 8 showed a significant decrease in pH in patients with AD compared with the corresponding controls within each dataset (GSE44771, *P* = 3.51 × 10^−7^; GSE44770, *P* = 1.22 × 10^−5^; GSE44788, *P* = 1.10 × 10^−4^; GSE84422, *P* = .044; Guennewig 2021, *P* = .0016; Haytural 2021, *P* = .015; Jacobs 2006, *P* = .021; Passila 2019, 2020, *P* = .033; 2-tailed unpaired *t* test). The others showed no significant difference in pH between patients and controls. No datasets showed a significant increase in pH. Funnel plot and Egger test showed no evidence of publication bias for the 20 datasets (*t *= −0.95, *P* = .35; supplementary Figure 1). The meta-analysis using the random-effects model revealed a significant decrease in pH in patients with AD compared with control subjects (Hedges’ *g *= −0.53, *z* = −5.70, *P* < .0001, 95% CI = [−0.72; −0.35]) (Figure 2). The *I*^*2*^ test showed high heterogeneity, suggesting significant differences between datasets (*I*^*2*^ = 59.0%, *P* < .01). In a 2-way ANCOVA with the dataset and diagnosis as the main factors, the effect of diagnosis remained significant (mean square [MS] = 2.34, *F* = 24.60, *P* < .0001), while no significant effects were observed for PMI (MS = 0.11, *F* = 1.14, *P* = .29), age at death (MS = 0.33, *F* = 3.50, *P* = .062), or sex (MS = 0.24, *F* = 2.56, *P* = .11). The effect of dataset was significant (MS = 1.23, *F* = 12.91, *P* < .0001). These results suggest that, while the pH varied between datasets, it was decreased in patients with AD compared with controls, independently of the covariates examined.

### Effect of Sample Type, Brain Region, and Tissue Integrity on pH Changes

The 8 datasets mentioned above that showed significant differences in pH in individual datasets were all based on brain samples. Subgroup analysis by sample type revealed a significant decrease in pH in brain samples of patients with AD compared with controls (*P* < .0001, 17 datasets) and a trend toward a decrease in CSF samples (*P* = .095, 3 datasets) (supplementary Figure 2A and B). Considering brain regions, we conducted subgroup analyses on datasets where the brain regions examined for pH were clearly specified in the original studies. The results showed a significant decrease in pH in patients with AD compared with controls in both cerebral cortical samples (*P* < .0001, 3 datasets) and cerebellar samples (*P* < .0001, 3 datasets) (supplementary Figure 2C and D).

Tissue pH is often considered a quality-control indicator for postmortem brain tissues due to its significant positive correlation with RNA integrity number (RIN), where low RIN indicates poor RNA quality (Durrenberger et al., 2010; Robinson et al., 2016). However, some studies report no significant or even negative correlations (White et al., 2018; Ono et al., 2019), indicating that this idea remains controversial. In this study, we observed a significant decrease in RIN in patient samples compared with controls in 3 of the 5 datasets reporting RIN data (supplementary Figure 3A–E). Furthermore, there was a significant positive correlation between pH levels and RIN (supplementary Figure 3F), suggesting that tissue integrity may be a potential confounding factor for pH measurements in this study.

### A Decrease in pH Is Associated With Progressive Brain Pathological Changes

The current assessment of the severity of pathological changes in AD is mainly based on the Braak staging, where stages I–II represent clinically silent cases, stages III–IV incipient AD, and stages V–VI fully developed AD (Braak and Braak, 1991). We then examined the relationship between brain pH levels and the Braak stage. In the 15 datasets obtained (supplementary Table 1), 4 showed a significant negative correlation between pH levels and Braak stage in each dataset (GSE84422, *r* = −0.24, *P* = .015; Passila 2019, 2020, *r* = −0.50, *P* = .018; Jacobs 2006, *r* = −0.49, *P* = .0064; Haytural 2021, *r* = −0.64, *P* = .010). The others showed no significant correlation within datasets. The meta-analysis, employing a random-effects model, revealed a significant negative correlation between pH levels and Braak stage (adjusted *r* = −0.26, *P* < .0001, 95% CI = [−0.38; −0.13]) (Figure 3). This negative correlation was corroborated by linear regression analysis on 15 combined datasets, standardized using z-score normalization, which showed a modest but significant relationship (*r* = −0.20, *P* = 1.37 × 10^−5^; supplementary Figure 4). Furthermore, a similar negative correlation between brain pH levels and scores for amyloid pathology was observed in a meta-analysis of 8 datasets, assessed using a random-effects model (adjusted *r* = −0.18, *P* = .0076, 95% CI = [−0.31; −0.048]; supplementary Figure 5). These results suggest that lower pH levels are associated with higher severity of the disease.

The brain pH also showed significant negative correlations with other pathological and clinical rating scores, including clinical dementia rating (*r* = −0.22, *P* = .026), average neuritic plaque density (*r* = −0.25, *P* = .0095), sum of Consortium to Establish a Registry for Alzheimer’s Disease rating scores in multiple brain regions (*r* = −0.34, *P* = .0004), and sum of neurofibrillary tangles density in multiple brain regions (*r* = −0.24, *P* = .014) (supplementary Figure 6). These results support the idea that lower pH levels are associated with higher severity of the disease.

### Brain pH Improves Discriminative Ability of *APOE*ε4 Status in AD

The ε4 allele of *APOE* is the strongest genetic risk factor for sporadic AD (Belloy et al., 2019). While *APOE*ε4 status did not affect brain pH level either in patients with AD or control subjects (supplementary Figure 7), it exhibited a significant discriminative ability in identifying patients from controls, as determined by the ROC curve analysis using 7 datasets consisting of 264 subjects (Figure 4; supplementary Table 1). The ROC AUC values of the single-predictor model were 0.66 for *APOE*ε4 status (95% CI: [0.60; 0.73], *P* < .0001) and 0.62 for the pH level (95% CI: [0.55; 0.69], *P* = .0008). When the pH level was added to the *APOE*ε4 status, the ROC AUC value increased to 0.72 (95% CI: [0.66; 0.78], *P* < .0001; *P*_difference_ = .0075) (Figure 4). The discriminative accuracy of *APOE*ε4 status was improved by the combination with the pH level, suggesting that these 2 variables have a complementary value.

## DISCUSSION

The present study suggested that brain pH is decreased in patients with AD compared with control subjects even when PMI, age, and sex were considered as potential confounding factors. We recently showed that a decrease in brain pH in AD can be estimated through the analysis of gene expression patterns (Hagihara et al., 2023), which aligns with the findings of this study. Furthermore, the increased levels of brain lactate in patients with AD provide additional support for the observed decrease in pH.

A decrease in brain pH has been linked to an increase in lactate levels in schizophrenia and bipolar disorder (Prabakaran et al., 2004; Stork and Renshaw, 2005; Halim et al., 2008). The simultaneous decrease in brain pH and increase in lactate levels observed in the same samples from a well-known AD mouse model harboring the human amyloid precursor protein mutant allele, as well as in various animal models of other neuropsychiatric disorders, further highlight their relationship to metabolic changes in AD and other disorders (Hagihara et al., 2024). An increase in lactate levels is considered indicative of metabolic alterations resulting from mitochondrial dysfunction and/or increased glycolysis due to neuronal hyperexcitation. Lactate is thought to be a relatively strong acid due to its ability to almost completely dissociate into H^+^ ions and lactate anions at cellular pH (Siesjö, 1985). Indeed, hyperexcitation in certain neurons and/or neural circuits has been suggested in the etiology of AD (Busche and Konnerth, 2016; Murano et al., 2019; Maestú et al., 2021). Furthermore, an increase in brain (Mullins et al., 2018; Hirata et al., 2024) and CSF (Liguori et al., 2015, 2016) lactate levels has been observed in patients with AD compared with control subjects. Neuronal hyperexcitation (Palop et al., 2007; You et al., 2017) along with increased brain lactate and decreased brain pH levels (Hagihara et al., 2024) have been observed in the aforementioned mouse model of AD. At a cellular level, glycolysis and subsequent lactate release are stimulated by the uptake of the neurotransmitter glutamate in astrocytes following neuronal activation, as demonstrated in an in vitro study (Pellerin and Magistretti, 1994). Astrocytes are considered to prefer lactate production in the brain. A shift toward excitation would increase energy demands in neurons, potentially prompting astrocytes to elevate lactate production, which in turn could lower brain pH. Indeed, increased lactate levels, accompanied by astrocyte activation, have been observed in the brains of individuals of AD (Hirata et al., 2024). Therefore, the observed decrease in brain pH may be attributed to elevated lactate levels induced by neuronal hyperexcitation in AD. Activity-dependent acidosis has been suggested to suppress neuronal activity in animal models of neonatal febrile seizures and is proposed as an intrinsic mechanism for the self-termination of seizures (Chesler and Kaila, 1992; Schuchmann et al., 2006; Pavlov et al., 2013). Further research is needed to determine whether a decrease in brain pH is related to such compensatory mechanisms in chronic degenerative diseases. Additionally, the role of other factors, such as the production of carbon dioxide—another metabolic acid regulated by neuronal activity—should be considered in the reduction of brain pH (Zauner et al., 1995; Chesler, 2003). While lactate is a key regulator of brain pH (Prabakaran et al., 2004), various other factors may also play a role in its decrease.

We first conducted a meta-analysis of pH levels comparing patients with AD and controls using mixed sample types, including postmortem brain samples (17 datasets) and CSF samples (3 datasets). Possibly due to the small sample size (51 patient and 28 control samples in total), the subsequent subgroup analysis showed a trend toward decreased pH in the CSF of patients compared with controls. A previous study reported a significant decrease in CSF pH in patients with AD in a larger cohort (Decker et al., 2021). As mentioned above, considering the increased lactate levels in the CSF of patients with AD (Liguori et al., 2015, 2016), these findings of decreased pH are promising. Although consistent results regarding decreased CSF pH have been observed in AD, the number of studies remains limited, highlighting the need for further research with additional cohorts.

We found that brain pH was negatively correlated with Braak stage and score for amyloid pathology, suggesting a lower brain pH as the disease course progresses. Braak staging of AD is based on the propagation of tau neurofibrillary tangles in the brain and the stage of tau pathology is associated with cognitive impairment (Braak and Braak, 1991). It is hypothesized that soluble amyloid-β oligomers cause hyperphosphorylation of tau and the accumulation of neurofibrillary tangle (Zheng et al., 2002); however, the local relationship between amyloid and tau was restricted in several brain regions (Iaccarino et al., 2018), suggesting additional causes for tau accumulation. Interestingly, neuronal hyperexcitation has been suggested to enhance tau protein translation in neurons, which may lead to the pathological accumulation of tau in soma and dendrite of neuronal cells (Kobayashi et al., 2017, 2019). Therefore, a decrease in pH and the accumulation of tau may occur in parallel due to neuronal hyperexcitation. Furthermore, acidic conditions could increase the abnormal hyperphosphorylation of tau protein in vitro (Basurto-Islas et al., 2013). Amyloid aggregation is also known to be aggravated under acidic conditions (Barrow and Zagorski, 1991; Burdick et al., 1992; Atwood et al., 1998; Su and Chang, 2001). Accumulation of amyloid-β also induces neural hyperexcitation (Zott et al., 2019; Maestú et al., 2021) and possibly vice versa (Yan et al., 2012). These potential direct and indirect relationships between pH changes and tau and amyloid pathologies, mediated by neural hyperexcitability, could contribute to the observed negative correlation of pH with Braak stage and amyloid pathology score. Brain pH may serve as a surrogate marker for these brain pathologies, although the causal relationship remains unclear.

We found that brain pH significantly improved discriminative accuracy of *APOE*ε4 status in distinguishing patients with AD from control subjects. However, the improved AUC value of 0.72 was still moderate, suggesting limited utility of their combination for diagnostic purposes. A previous in vivo MRS study showed that brain pH improved diagnostic accuracy of brain metabolite (i.e., N-acetylaspartate to creatine ratio) for AD (Lyros et al., 2020). While brain pH alone exhibited relatively low (AUC of 0.62 in this study) to moderate (AUC of 0.81 in Lyros et al., 2020) accuracy for distinguishing patients with AD from control subject, combining it—measured in CSF or by MRS—with multiple markers, such as a blood-based protein marker panel (Jiang et al., 2024), could be a valuable biological strategy to assist in the diagnosis of AD and the prediction of disease progression.

Regarding potential bias in this study, the Egger regression test on the funnel plot showed no significant publication bias across datasets analyzed. Additionally, relying on demographic rather than primary outcome data may minimize typical outcome bias. It is also important to consider whether the datasets analyzed in this study are broadly representative of the AD population. We observed no difference in pH between subpopulation of patients with or without the *APOE*ε4 allele. This result suggests that the datasets used in this analysis are unbiased from the perspective of *APOE*ε4 status, although biases from other perspectives cannot be ruled out.

A significant limitation of this study may involve the lack of consideration for other potential confounding factors affecting postmortem brain pH than age at death, PMI, and sex. Factors before death, or agonal states, and the cause and manner of death can influence postmortem brain pH through alterations in blood oxygenation, brain perfusion, and biochemical content (Lewis, 2002; Tomita et al., 2004). If patients with AD experienced prolonged agonal conditions compared with controls in the datasets used, we cannot rule out the possibility that the observed decrease in pH was influenced by such factors. Postmortem brain pH is also thought to be influenced by tissue integrity, although this notion remains controversial, as a high pH does not necessarily indicate intact tissue RNA (Webster, 2006). It is technically difficult to exclude the influence of potential confounding factors in human studies. In this circumstance, studies in animal models can be useful alternatives to confirm whether changes in brain pH are involved in the pathophysiology of neuropsychiatric disorders, as they are essentially exempt from such confounding factors. In our recent study, we showed that amyloid precursor protein transgenic AD model mice exhibited a decrease in brain pH compared with corresponding control mice (Hagihara et al., 2024), supporting the notion that this phenomenon is intrinsically related to pathophysiology of AD rather than mere an artifact.

In conclusion, this study suggested that postmortem brain and CSF pH is decreased in patients with AD compared with control subjects. The decrease in pH may occur independent of having *APOE*ε4 genotype, the most prominent genetic risk of AD. Neuronal hyperexcitation may lead to the decrease in pH, which may be exacerbated in the disease course. It remains undetermined whether such a decrease in pH is associated with either beneficial or detrimental effects on psychiatric conditions, which is required to be addressed in the future studies.

## Supplementary Material

pyae047_suppl_Supplementary_Figures

pyae047_suppl_Supplementary_Table_S1

pyae047_suppl_Supplementary_Table_S2

## Data Availability

The raw data analyzed in this study are provided in supplementary Table 2.

## Notes

This work was supported by MEXT Promotion of Distinctive Joint Research Center Program grant number JPMXP0618217663 and JSPS KAKENHI grant number JP20H00522.

## Interest Statement

The authors have nothing to disclose.

## Additional information

The review was not registered elsewhere. The review protocol was not prepared.
