# Supplementary material for: Decreased Brain pH Correlated With Progression of Alzheimer Disease Neuropathology: A Systematic Review and Meta-Analyses of Postmortem Studies
Source: Int J Neuropsychopharmacol. 2024 Oct 18;27(10):pyae047. doi: 10.1093/ijnp/pyae047 (PMC11511658; doi:10.1093/ijnp/pyae047)
Supplement: pyae047_suppl_Supplementary_Figures [file pyae047_suppl_supplementary_figures.docx]

**Supplementary Figures for**

**Decreased brain pH correlated with progression of Alzheimer’s disease neuropathology: a systematic review and meta-analyses of postmortem studies**

Hideo Hagihara^1^ and Tsuyoshi Miyakawa^1^

^1^Division of Systems Medical Science, Center for Medical Science, Fujita Health University, Toyoake, Japan

**Supplementary Figure 1. Funnel plot of the included datasets.** Each plot represents a dataset. SE, standard error; SMD, standardized mean difference.

**Supplementary Figure 2. Effect of sample type and brain region on changes in pH levels in AD.** Scatter plots of z-score-transformed pH values of brain (A), CSF (B), cerebral cortical (C), and cerebellar samples (D) of patients with AD and control subjects. A total of 1168 samples from 17 datasets (A), and 79 samples from three datasets (B), 530 samples from three datasets (C), and 284 samples from three datasets (D) were analyzed. The z-scores were calculated within each dataset. Red bars indicate median values for each group. The p-values from the unpaired t-test are shown.

**Supplementary Figure 3. Positive correlation between brain pH and RNA integrity number (RIN).** **A–E** Scatter plots of RIN in the datasets GSE44768 (A), GSE44770 (B), GSE44771 (C), Garamszegi 2023 (D), and Guennewig 2021 (E). Red bars indicate the median values for each group. The p-values from the unpaired t-test are shown. **F** Scatter plot showing the correlation between z-score-transformed pH values and RIN. The z-scores were calculated within each dataset. A total of 743 samples from five datasets were analyzed. *r*, Pearson’s correlation coefficient.

**Supplementary Figure 4. Scatter plot depicting the negative correlation between pH (z-score) and Braak stages.** A z-score was calculated for each subject within the dataset to standardize pH measurements. Each dot represents the data for an individual subject. *r*, Pearson correlation coefficient.

**Supplementary Figure 5.** **Negative correlation between brain pH and amyloid pathology scores.** Forest plot of meta-analysis of correlation between brain pH and amyloid stages. Adjusted *r* = -0.18, *p* = 0.0076, 95% CI = [-0.31; -0.048]. 95% CI, 95% confidence interval; CC, correlation coefficient; SE, standard error.

**Supplementary Figure 6. Significant correlation between a decrease in brain pH and an increase in disease severity.** Data of GSE84422 was analyzed. Scatter plot of correlation between brain pH and clinical dementia rating (A), average neuritic plaque density (B), sum of Consortium to Establish a Registry for Alzheimer's Disease (CERAD) rating scores in multiple brain regions (C), and sum of neurofibrillary tangles density in multiple brain regions (D). The solid line indicates the regression line, and the dashed line indicates the 95% confidence interval. *r*, Pearson correlation coefficient.

**Supplementary Figure 7. No significant effect of *APOE*ε4 status on brain pH.** Forest plot of meta-analysis comparing postmortem brain pH between *APOE*ε4 carriers and non-carriers in control subjects (A; Hedges’ *g* = -0.40, 95% CI = [-0.80; 0.0056], *p* = 0.053) and patients with AD (B; Hedges’ *g* = 0.046, 95% CI = [-0.31; 0.40], *p* = 0.80). 95% CI, 95% confidence interval; SE, standard error; SMD, standardized mean difference.
